# Platelet-Derived Procoagulant Microparticles as Blood-based Biomarker of Breast Cancer

**DOI:** 10.31557/APJCP.2021.22.5.1573

**Published:** 2021-05

**Authors:** Marzieh Haghbeen, Akbar Hashemi Tayer, Maryam Kamravan, Abdolreza Sotoodeh Jahromi

**Affiliations:** *Research Center for Noncommunicable Diseases, Jahrom University of Medical Sciences, Jahrom, Iran.*

**Keywords:** Breast cancer, microparticle, platelet, prognostic

## Abstract

**Objective:**

Breast cancer is the main cause of cancer death in women worldwide. Elevated plasma levels of circulating cell-derived microparticles (MPs) have been reported in various types of cancer, including breast cancer, with the ability to mediate inflammation and thrombosis. Microparticles are bioactive agents, and it has been suggested that MPs can be used as a diagnostic, prognostic, or therapeutic biomarker in various diseases. The aim of this study was to investigate the levels of platelet-derived MPs (PMPs) in breast cancer patients.

**Materials and Methods:**

In this case-control study, 30 patients with breast cancer and 20 normal subjects were sampled after obtaining written consent. MPs were isolated from blood samples by centrifugation technique. CD42b and annexin V markers were used respectively for counting PMPs and procoagulant MPs with flow cytometry.

**Results:**

Flow cytometry results showed that the number of PMPs and procoagulant annexin V positive MPs was significantly higher in the breast cancer patients than normal subjects (p<0.001). The number of the annexin V MPs differed significantly in patients with high tumor size (T2) compared to the patients with low tumor size (T1) and controls (p<0.001). Significant and positive correlations were found between PMP levels and tissue-based biomarkers, tumor grading, and distant metastasis (P<0.05). Tumor histological type did not correlate with the numbers of PMPs (p=0.065).

**Conclusion:**

Increased levels of PMPs and activity in terms of hemostasis and having a positive and significant relationship with tumor grading and metastasis may indicate the effective role of PMPs in the pathogenesis and prognosis of breast cancer.

## Introduction

Breast cancer is the most frequently diagnosed invasive cancer in women worldwide (Li et al., 2020), and also is the second cause of cancer mortality after lung cancer (Siegel et al., 2019). Accurate early screening of breast cancer is critical in the management of the patients and there is an urgent need to develop novel biomarkers for early diagnosis of breast cancer (Zubor et al., 2019). To identify breast cancer patients, compared to invasive diagnostic approach including core biopsy, the use of blood biomarkers is not only convenient and non-invasive, but also widely accepted, cost-effective, and readily reproducible (Li et al., 2020). The most useful tissue-based biomarkers in breast cancer include estrogen receptor (ER), progesterone receptor (PR), human epidermal growth factor receptor 2 (Her-2), and ki67 are used to detect prognosis and guiding systemic therapy. In addition, breast cancer antigens (BRCA), urokinase plasminogen activator (uPA), carcinoembryonic antigen (CEA), and cancer antigen (CA)15-3, are used as blood-based biomarkers in breast cancer (Duffy et al., 2017; Duffy et al., 2016). The main applications of CA15-3 and CEA as the most used blood biomarkers are to predict response to therapeutic regimens in breast cancer patients with metastasis, as well as for assessing prognosis (Toth et al., 2008). Over the last decade, many research efforts have been focused on the development of non-invasive blood-based biomarkers for breast cancer diagnosis at an earlier stage.

Circulating extracellular microparticles in the peripheral blood have emerged as a potential non-invasive biomarker to supplement current clinical approaches that allow earlier detection of cancer. Some reports have indicated that cancer patients possess high levels of circulating procoagulant MPs and an increased risk of thrombosis (Rak, 2010; Zwicker, 2010). Microparticles (MPs) are membrane-derived small vesicles (0.1- 1.0 µm) that are released from the surface of various cells including platelets, red blood cells (RBC), leukocytes, and endothelial cells during cellular activation, and apoptosis (Melki et al., 2017; Westerman and Porter, 2016). MPs contain bioactive anionic phospholipids (phosphatidylserine; PS), cytoplasmic components, and various antigens that are characteristic of the state of the originating cells (Morel et al., 2011). The expression of PS plays a key role in the MPs generation process and is also required for the assembly of coagulation enzyme complexes, subsequently enhance the thrombin generation (Nébor et al., 2013). Furthermore, MPs are considered to have other biological roles in vascular function, angiogenesis, and inflammation (Kent et al., 2014; Rubin et al., 2013). 

MPs are present at a low level in the peripheral blood of healthy individuals; where they originate mainly from platelets (Berckmans et al., 2001). On the other hand, they are elevated in many clinical disorders such as diabetes (Rodrigues et al., 2018), sickle cell anemia (Lapping-Carr et al., 2020), cardiovascular disease (Van der Pol et al., 2012), and in different kinds of malignancies including lung (Wang et al., 2017), gastric (Wang et al., 2018), colorectal cancer (Wojtukiewicz et al., 2020), leukemia (Nafady et al., 2018), lymphomas (Savaşan et al., 2004), pancreatic (Faille et al., 2018) and breast cancer (Zarà et al., 2017; Zahran et al., 2021). 

Platelet-, monocyte-, and endothelial-derived MPs are the most frequently detected MPs in cancer patients (Janowska-Wieczorek et al., 2006). Many roles of circulating MPs in cancer progression have been clarified, including the following: angiogenesis, tumor growth through interactions with circulating tumor cells, accelerate chemotaxis, escape from immune surveillance and apoptosis, and chemoresistance (Mezouar et al., 2014). Additionally, PMPs promote metastasis and angiogenesis in lung cancer patients (Janowska-Wieczorek et al., 2005). In patients with prostate cancer, a high level of PMPs is correlated with short survival (Helley et al., 2009). There is evidence indicating that MPs may be a useful prognostic and diagnostic biomarker of breast cancer (Mezouar et al., 2014).

The aim of this prospective case–control study was to determine the circulating procoagulant PMPs levels as biomarkers in women with breast cancer. Therefore, MPs-expressing CD42b, procoagulant MPs, and tissue-based tumor markers were evaluated in breast cancer patients. 

## Materials and Methods

*Patient characteristics*

The study and consent procedures were approved by The Ethics Committee for The Jahrom University of Medical Sciences (IR.JUMS.REC.1397.091). In this case-control study, 30 newly diagnosed patients with histologically confirmed breast cancer, and 20 age-matched healthy volunteers who did not have any known coagulation disorder were included. The patients were classified according to the tumor size, lymph node, and metastasis (TNM) system. The healthy controls had mammograms without abnormal pathological findings within the last year, and did not smoke. Afterward, written consent of the patients and healthy volunteers were received after the procedure was explained to them in detail. In this study, exclusion criteria including the following; chemotherapy within the last 6 months, anticoagulant therapy, thrombotic events in the last year, uncontrollable chronic diseases, smoking, and taking oral contraceptives.

*Blood sample and measurements*

Whole blood (10 ml) was obtained through clean phlebotomy of an antecubital vein using 20-gauge needles (BD Vacutainer needles). The status of estrogen receptor (ER), progesterone receptor (PR), and human epidermal growth factor receptor 2 (Her-2) were determined by conventional immunohistochemistry upon the formalin-fixed paraffin-embedded blocks of breast carcinoma patients according to current guidelines. Additionally, complete blood count (CBC) was determined in the whole study population using an automated hematology analyzer (Sysmex XT 2000i, Diamond Diagnostics, USA) according to the manufacturer’s recommendations. 

*MPs isolation and purification*

Samples were evaluated for the presence of PMPs. At first, MPs were isolated from the plasma with centrifugation. For this purpose, 5 ml of plasma was mixed with 1 ml of PBS (pH 7.4) and centrifuged at 2,000×g for 10 minutes at 10°C. The supernatant was centrifuged again with this protocol to exclude residual debris. The centrifuge brake was set to “off” to avoid mixing cells and debris with the supernatant. The top two-thirds of the double centrifuged plasma was then removed and stored at -70°C until analysis. 

*Quantification of MPs by flow cytometry*

In this study, Flow cytometric analysis was conducted with cy flow space flow cytometer (Partec PAS, Germany) using Flomax software, as previously described (Hashemi Tayer et al., 2019). Flow cytometric analysis was performed with a precise number of beads to determine MPs count, and specific conjugated antibodies were used to identify the origin cell of the MPs. Using Fluoresbrite® Carboxylate Microspheres 1.0 µm (Polysciences, Warrington, Philadelphia), size events were defined. 

Phycoerythrin (PE) anti-human CD42b (BD Pharmingen, San Diego, CA, USA) was used to tag PMPs. Moreover, we used fluorescein isothiocyanate (FITC) human annexin V (BD), which tags PS on procoagulant MPs. PE Mouse IgG2b k isotype control (BD) was also used as a negative control to differentiate the background noise of the cytometric analysis.

The cellular origin and concentration of PMPs were determined immediately as follow; Samples containing MPs (50 µl) were labeled with anti-CD42b (5 µl) and PE-IgG2b isotype control (5 µl) for 30 minutes at 4°C in the dark. Also, 50 µl of samples were added to test tubes containing 300 µl of binding buffer (0.1M HEPES/pH 7.4, 25 mM CaCl_2_, and 1.4 M NaCl), and then conjugated FITC-AnnV (5 µl) was added. After 30 minutes of incubation at 22°C in the dark, samples were analyzed with a cytometer. Before analysis, 5µl of well-mixed 1.0 µm beads that were diluted 1/500 in double-distilled water was added to each sample (Hashemi Tayer et al., 2019). The concentration of PMPs was calculated by comparison to the bead concentration. The number of PMPs was measured in relation to ten thousand beads events. In order to discriminate true events from the background noise, PMPs were defined by size (less than 1.0 μm), platelet origin (CD42b), and PS exposure (AnnV positive) as previously described (Ayers et al., 2011; Grisendi et al., 2015). The absolute count of MPs per microliter was calculated as follow:

MPs per µl=(N.of events in gating containing MPs×Absolute count of bead per tube)/(N.of events in bead region×test volume) 

*Statistical analysis*

Data are presented as mean and standard deviation (SD). Repeated measure ANOVA, paired t-test, and Pearson correlation were used for data analysis. Stata software, version 13 (Stata Corp, College Station, TX, USA) was used for all statistical analysis. Graphs were depicted by Excel and Stata software. Statistical significant differences were considered as p less than 0.05.

## Results

*Study population*

The results showed that the mean age of the breast cancer patients was 50.4±11.08 (31-73) years and did not differ significantly from the healthy controls (49.2±9.6 (29-64) years). The body mass index (BMI), CBC indices, and also coagulation screening tests did not differ significantly between case and control groups (P>0.05; Table1). 

The patients were classified according to the TNM system: 7 patients had tumors <2 cm (T1) and 23 had tumors >2 cm (T2). 17 patients had negative lymph nodes (N0), and 13 had positive lymph nodes involvement (N1). 9 patients had negative metastasis (M0), and 21 patients had positive organ involvement (M1). Based on the histological category; 20 patients had criteria for invasive ductal carcinoma, 4 patients had ductal carcinoma in situ, and 5 patients had lobular carcinoma. About tissue-based biomarkers, 2 patients were positive for ER, 21 for PR, and 18 patients were positive for Her-2. The clinicopathological data of all patients with breast cancer are given in Table 2.

*MPs analysis*

**Figure 1:**
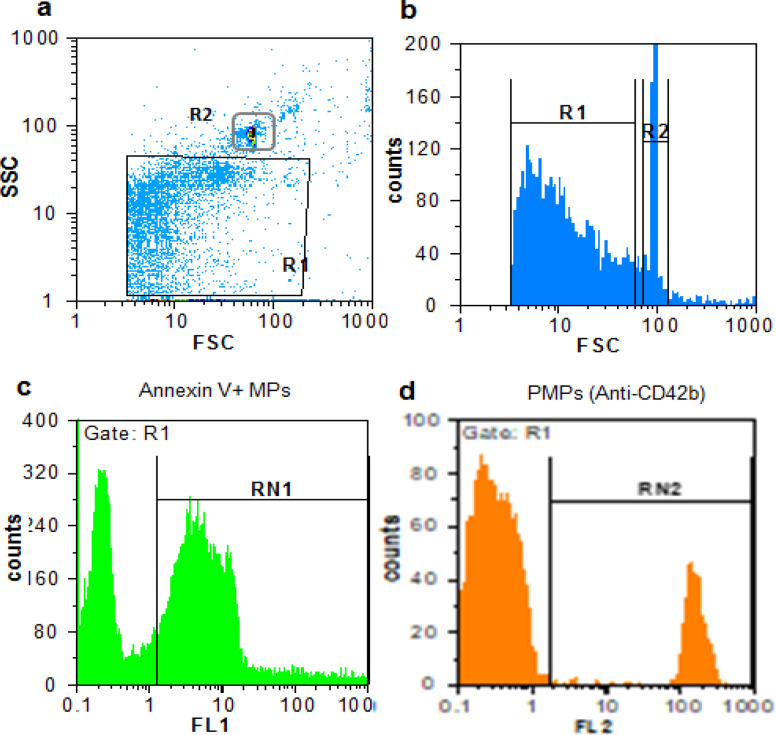
Flow Cytometry Plots of PMPs in Breast Cancer Patients. Forward scatter (FSC) and sideward scatter (SSC) indicates respectively size and granularity. The MPs size gate was set by 1.0 μm beads. a) Two gating regions are region R1 and R2 that represents MPs and 1.0 μm beads, respectively. Based on the FSC and SSC, PMPs are located lower than 1.0 µm beads. b) A histogram of logarithmic forward scatter versus MPs count, showing the distribution of MPs in comparison to the beads. c) Region RN1 represents region R1 events that were labeled with conjugated FITC-annexin V. d) Region RN2 represents region R1 events that were labeled with conjugated PE anti- CD42b and indicates PMPs. Non-stained events are demonstrated by the left peak in Figure c-d

Flow cytometric analysis of all subjects revealed that all cases and control samples tested had PMPs. First, MPs were gated on the basis of their specific FSC and SSC pattern (Figure1a, 1b). As shown, MPs were seen as the population that had lower FSC and SSC than the 1.0 µm beads. Afterward, MPs were further identified by staining with fluorescent annexin V (Figure 1c), and CD42b (Figure1d) markers.

*CD42-positive microparticles*

The total number of MPs, as well as two measured subpopulations, were significantly elevated in breast cancer patients compared to the controls.

**Figure 2:**
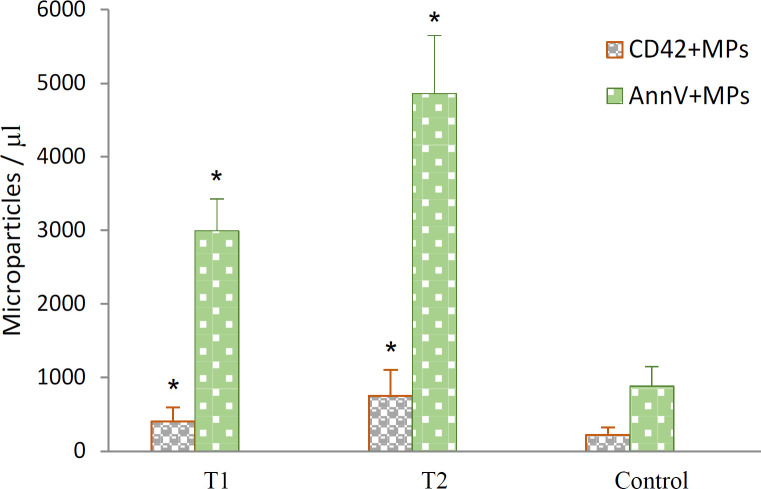
The Number of PMPs (CD42b+) and Procoagulant MPs (annexin V+) in Breast Cancer Patients (T1, and T2 groups) and Control Groups. * Significant difference (p < 0.001) between patients and control groups, by paired t-test. Results are presented as mean±standard deviation

Considering tumor size status and PMPs count, significantly higher PMPs levels were found in patients with T1 and T2 compared to the control group (2.6-fold; p<0.001) (Figure 2).

Patients with larger tumor size (T2) had significantly higher concentrations of PMPs (750±356/ µl; p<0.001), than patients with smaller tumor size (T1) (401.1±198.8/µl) and controls (217.4±108.6/µl) (Figure 2). As reported in previous studies, there was a significant variation of the MPs counted between samples that were obtained from different volunteers (Hashemi Tayer et al., 2019; Ayers et al., 2011)

*Annexin V positive microparticles*

 As expected, the MPs were also positive for annexin V staining (61%, Figure 1c). Annexin V is a useful marker to determine the general levels of procoagulant MPs. A wide range in the numbers of circulating annexin V+ MPs was found in breast cancer patients and controls. The breast cancer patients had higher annexin V+ MPs levels compared to the controls (4.46-fold; p<0.001) (Figure 2).

The number of the annexin V+ MPs differed significantly in patients with high tumor size (T2) (4858±789/µl; p<0.001) compared to the patients with low tumor size (T1) (2995.8±439.2/µl) and controls (880±269.6/µl) (Figure 2). The changes in the number of annexin V+ MPs correlated with the number of CD42+ MPs (r= 0.996; P<0.001 by Pearson’s test). These data indicated that the changes in the number of PMPs and annexin V+ MPs may be associated with breast cancer. 

*Correlation analysis*

Pearson correlation analysis was carried out to determine the relationship between the concentrations of PMPs with tissue-based biomarkers, tumor grade, tumor histological type, and tumor metastasis. PMPs concentration strongly correlated with both tumor grade (*r* = 0.92; *p*< 0.001) and metastasis (*r* = 0.96; *p*< 0.001). Tissue-based biomarkers were also significantly associated with PMPs count (ER: *r *= 0.53, *p*< 0.04; PR: *r* = 0.43, *p*< 0.03, Her-2: *r* = 0.52, *p*< 0.031), whereas tumor histological type and numbers of PMPs (CD42b+) did not correlate (*r* = 0.28, *p*< 0.065) (Table 3).

**Table 1.** Study Population. Parametrically distributed data are presented as mean± standard deviation (minimum-maximum).

Parameters	‌Breast Cancer (N=30)	Control (N=20)	P-value
Age (year)	50.4±11.08 (31-73)	49.2± 9.6 (29-64)	Ns
BMI (kg/m^2^)	23.4±3.5 (17.1-31.4)	24.9±4.4 (16.1-35.3)	Ns
Hb (gr/dl)	12.1±1.40 (8.7-14)	13.1±1.2 (10-14.1)	Ns
HCT (%)	37.1±4.70 (25-43)	39.3 ±5.3 (31-44)	Ns
MCV (fl)	80.0±6.9 (61-91)	82.1±5.8 (69-95)	Ns
MCH (pg)	26.1±2.7 (19-31)	26.2±2.5 (21-30)	Ns
RBC (×10^6^/µl)	4.6±0.91 (3.8-6.1)	4.7±0.88 (3.7-5.8)	Ns
Leukocyte (×10^3^/µl)	6.8±2.60 (4.1-14.3)	7.2±1.9 (3.9-12.2)	Ns
Platelet (×10^3^/µl)	232.8±63 (131-370)	241±72 (142-395)	Ns
PT (sec)	11.8±0.85 (11-13)	11.4±0.78 (11-13)	Ns
PTT (sec)	34.3±4.90 (26-44)	33.6±3.5 (25-40)	Ns

Hb, hemoglobin; HCT, hematocrit; MCV, mean corpuscular volume; MCH, mean corpuscular hemoglobin; RBC, red blood cell; PT, prothrombin time; PTT, partial thromboplastin time; Ns, not significant

**Table 2:** Baseline Characteristics of the Studied Breast Cancer Patients

N=30	‌Breast Cancer Patients	
	N	%
Tumor size		
T1	7	23.33
T2	23	76.70
Node		
N0	17	57.00
N1	13	43.00
Metastasis		
M0	21	70.00
M1	9	30.00
Tumor Grading		
1	2	6.66
2	12	40.00
3	16	53.33
Histologic type		
Ductal Carcinoma In Situ	4	13.33
Lobular Carcinoma	5	16.66
Invasive Ductal Carcinoma	20	66.66
Paget's Disease	1	3.33
ER status		
Positive	25	83.00
Negative	5	17.00
PR status		
Positive	21	70.00
Negative	9	30.00
Her-2 status		
Positive	18	60.00
Negative	12	40.00

**Table 3:** The Correlation between Platelet-Derived Microparticles and Prognostic Indicators in Breast Cancer patients; Correlation Coefficient (r).

Prognostic indicator‌	Platelet-derived Microparticles	
	P-value	r
Her-2	0.031	0.52
ER	0.04	0.53
PR	0.03	0.43
Tumor Grade	0.001	0.92
Histologic Type	0.65	0.28
Tumor metastasis	0.001	0.96

## Discussion

Breast cancer is the second most commonly determined cancer in women, with an increasing incidence in developing countries. Despite the recent advances forward in diagnosis and treatment, cancer in most patients eventually progressed into distant metastasis. Owing to the fact that metastasis is the main cause of cancer death, new non-invasive biomarkers are needed for the early diagnosis of breast cancer (Siegel et al., 2019). The clinical relevance of MPs has been increasingly identified, emphasizing the urgent need for a better understanding of MPs biological activity and their roles in various malignancies (van der Pol et al., 2012). Although a wide variety of techniques are developed to identify MPs, generally there is no gold standard method to characterize them (Lacroix and Dignat-George., 2012). Based on several studies, flow cytometry is the most commonly used method for the assessment of the cell origin and enumerating MPs (Ayers et al., 2011; Grisendi et al., 2015).

In this report, we determined if circulating levels of procoagulant PMPs could predict clinical consequences in women patients with breast cancer. We evaluated 30 women patients at the initial diagnosis of breast cancer to identify and monitor circulating PMPs. In our study, patients and controls did not differ significantly concerning age, BMI, hemoglobin level, RBC count, platelet count, and leukocyte count. Despite the relatively small size of the study population, our finding showed considerable differences in the number of PMPs in breast cancer patients.

In this work, MPs were detected using flow cytometry on the basis of their size and density and verified using 1.0 µm standard beads, and then identified with specific conjugated antibodies. It seems that MPs events distributed near to the instrumental noise and debris (Macey et al., 2011). It should be noted that the small size of PMPs is the main limitation that challenges the reproducibility of flow cytometry technique, so when using this method, a limited number of MPs might be calculated (Nielsen et al., 2014).

In this study, we observed that circulating levels of the PMP were higher in breast cancer patients than in healthy controls, increasing levels with augmenting tumor size, and they were highest in patients with metastasis and lymph node involvement. Considerable higher PMP levels were found in patients with T2 compared to the T1 patients and control groups. Furthermore, we found statistical associations between PMP levels with tumor grade, metastasis, and also tissue-based tumor markers (ER, PR, and Her-2). These findings are in agreement with Toth (2008) study, which reported that PMP levels were significantly higher in breast cancer patients compared to the control samples, and these levels were significantly correlated with the presence of tumor metastasis. The authors showed a correlation of MPs levels with tumor grade and the leukocyte count.

Furthermore, Tesselaar (2007) observed significantly increased PMP levels in metastatic breast cancer and pancreatic cancer patients compared to healthy subjects. Variations of circulating levels of PMP have been reported according to the tumor grade and disease status.

With regard to PMPs, some authors reported significant increases in this MPs subpopulation in various malignancies (Mezouar et al., 2014b). Varon (2009) revealed that elevated PMP levels are significantly correlated with progressive tumors and poor prognosis. Helley (2009) reported a significant correlation between PMP levels and a median survival rate that was significantly shorter for patients with higher PMP levels compared to those with lower PMPs. Noteworthy, PMPs count varied significantly between patients’ samples (p<0.001). Variations in the concentration of PMPs were also observed between healthy volunteers. The precise reason for this variation remains understood, but it appears that several factors like ABO antigens, age, and sex have roles (Ayers et al., 2011; Rubin et al., 2010). 

Increased level of PMPs that are associated with increased thrombotic complications has been reported in several cancers (Chew et al., 2006). One of the key mechanisms contributing to the hypercoagulable state could arise from the cell membrane change and subsequent MPs formation (Provost, 2017). Expression of PS on the outer leaflet changes the cell membrane charge into a negative and provides a site to carry important procoagulant factors and support prothrombotic events (Morel et al., 2011; Nébor et al., 2013). Exposure of PS not only facilitates the formation of coagulation complexes, but also enhances the ability of tissue factor (TF) to initiate thrombin formation (Dinkla et al., 2014; Melki et al., 2017). 

For measuring the level of procoagulant MPs, FITC-annexin V was used that binds to PS. In our assay, flow cytometry analysis showed that the breast cancer patients had higher procoagulant MPs levels compared to the controls (p<0.001). The procoagulant MPs levels were elevated significantly in patients with high tumor size (T2) compared to patients with low tumor size (T1) and controls. 

However, these findings were contrary to the report by Liebhardt et al (Liebhardt et al., 2010), which concluded that breast cancer patients with smaller tumor size (T1) had a significantly higher level of annexin V+ MPs than controls, and also MP levels did not differ between T2 patients and controls.

The enrichment of PS and TF in the surface of PMPs has the main role in tumor progression-associated thrombosis. In our study, MPs membranes were enriched with PS, and based on this point, PMPs could support coagulant activities. As stated before, PMPs exhibit 50- to 100-fold higher specific procoagulant activity than activated platelets (Sinauridze et al., 2007), and it was suggested that high number of PMPs may induce the risk of thrombotic complications (Van Der Meijden et al., 2012). This data is consistent with previous observation indicating that patients with different cancers with or without thromboembolism presented significantly higher levels of PMPs and procoagulant MPs than the controls (Campello et al., 2011). Despite limited studies evaluating the role of PMPs in breast cancer, the identification of procoagulant PMPs is of great interest in cancer as a potential prognostic biomarker.

In conclusion, our findings suggest differences in the concentration of circulating procoagulant PMPs between breast cancer patients and controls, and also revealed the positive relationships between PMPs levels with tumor grade, and metastasis. Thus, the elevation of blood concentration of PMPs may be a valuable biomarker of poor prognosis in breast cancer. Taken together, further investigations will be needed to understand the possible role of MPs as a blood-based prognostic biomarker in the presence of a larger patient group, including patients with node involvement and metastasis.
